# Mitral Valve Aneurysm With Perforation Resulting in Severe Mitral Regurgitation Secondary to Infective Endocarditis: A Report of a Rare Case

**DOI:** 10.7759/cureus.93159

**Published:** 2025-09-24

**Authors:** Muhammad Usman Khalid, Tanveer Alam, Safia W Khan, Tania Carvalho, Alexandros Papachristidis

**Affiliations:** 1 Cardiology, King's College Hospital NHS Foundation Trust, London, GBR; 2 Medicine, Northwick Park Hospital, London, GBR

**Keywords:** infective endocarditis, mitral leaflet, mitral regurgitation, mitral valve aneurysm, perforation, transthoracic and transesophageal echocardiography

## Abstract

Mitral valve aneurysm (MVA) is a localised bulge of the mitral leaflet. Weakening of the mitral leaflet may be induced by infective endocarditis (IE), rheumatic disease, and connective tissue disorders like osteogenesis imperfecta, Marfan syndrome, and pseudoxanthoma elasticum. Complications of MVA include expansion, perforation, and regurgitation. IE, characterised by inflammation of endocardium, has significant morbidity and mortality. We report a case of mitral valve aneurysm with perforation in the setting of IE, diagnosed by transesophageal echocardiography (TEE). A 72-year-old male with multiple comorbidities presented with fluid overload and renal dysfunction. He had been previously admitted for sepsis, wet gangrene, and atrial fibrillation, which were treated medically. Blood cultures at the time grew *Staphylococcus aureus*. Upon investigating, a transthoracic echocardiogram (TTE) revealed multiple echogenic structures attached to the posterior mitral valve leaflet, likely consistent with vegetations. TEE revealed a calcified posterior mitral valve leaflet (PMVL) with an aneurysm along with perforation, resulting in severe mitral regurgitation, secondary to IE. Following multidisciplinary team (MDT) discussion, the patient was treated with antibiotics (intravenous followed by oral) due to multiple comorbidities with intravenous flucloxacillin for six weeks, followed by oral antibiotics, under the supervision of the outpatient parenteral antimicrobial therapy team. The patient completed the course with regular review by district nurses and the IE MDT, and remained clinically stable with no recurrence of bacteremia. TEE is considered superior to TTE in delineating the diagnosis of mitral valve perforation, as the former allows clear visualisation of complex mitral valve lesions. Prompt recognition and timely management are crucial in the prevention of mortality in patients with IE leading to perforation.

## Introduction

Mitral valve aneurysm (MVA) is a discrete protrusion of the mitral leaflet towards the left atrium with systolic expansion and diastolic collapse. Persistent MVA may result in perforation and rupture of an aneurysm, causing mitral regurgitation [1]. Its formation is associated with congenital conditions like left ventricular outflow tract obstruction (LVOTO), connective tissue disorders like Marfan’s syndrome, structural causes like mitral valve prolapse, as well as acquired factors like rheumatic fever, Libman-Sachs endocarditis, and infective endocarditis (IE) [2].

IE refers to the colonization of cardiac valve endocardium by virulent microorganisms (more often bacterial) [3]. Patients may present with a spectrum of symptoms from chest pain and dyspnoea to cardiogenic shock and hemodynamic instability [4]. Despite advances in clinical practice, timely diagnosis and adequate management of IE remain challenging. Outcomes are generally more favorable with surgical repair or replacement in suitable patients, while conservative treatment with antibiotics is reserved for those with prohibitive surgical risk [5]. Patients with conservatively managed IE and structural complications such as aneurysm or perforation should undergo repeat transesophageal echocardiography (TEE) at the end of therapy to confirm resolution of infection and reassess valve integrity [6]. This case highlights the importance of TEE in better visualisation of a complex condition, such as valve perforation, and management options for cases where surgical treatment is deemed unsuitable.

## Case presentation

A 72-year-old man with diabetes, hypertension, and a background of end-stage renal failure (ESRF), who was on peritoneal dialysis (PD) for the last two years, presented with fluid overload. He had recently returned from Sri Lanka after a two-week stay, during which he was admitted for urosepsis, left second toe amputation due to wet gangrene, and atrial fibrillation treated with amiodarone. Blood cultures obtained at the time grew methicillin-sensitive *Staphylococcus aureus* (MSSA), and the patient was treated with erythromycin, clindamycin, and flucloxacillin. After treatment, he was discharged with a plan for cardiology, nephrology, and vascular follow-up.

Upon return to the United Kingdom, he presented to A&E (Accident and Emergency) with dyspnoea and bilateral pitting oedema. His observations showed oxygen saturation at room air > 94%, blood pressure of 117/60 mmHg, and heart rate of 70 beats per minute. Examination revealed widespread crepitations throughout the lungs. Heart sounds were normal (HS I + II), with a grade III/IV pansystolic murmur at the apex of the heart radiating to the left axilla. Bilateral pitting oedema was noted, extending to the mid-shin. A PD catheter was seen in the left lower abdominal quadrant (LLQ). These findings were consistent with fluid overload and renal dysfunction.

Upon admission, the patient was initially treated for PD peritonitis, given a high white cell count in PD fluid. Laboratory investigations revealed elevated C-reactive Protein (61 mg/L), and serum creatinine (921 mmol/L). On physical examination by the diabetic team, healing of the left second toe amputation site was noted with no signs of soft tissue infection at the lateral heel blister. X-ray showed no evidence of osteomyelitis. 

**Figure 1:**
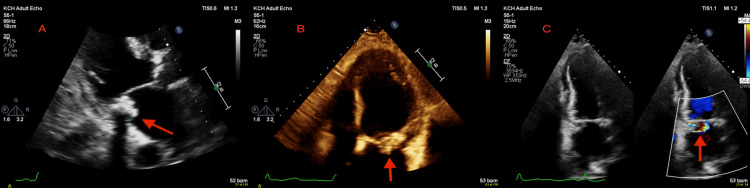
Transthoracic echocardiogram (A) Parasternal long-axis view showing echogenic structures attached to the posterior mitral valve leaflet extending from annulus to posterior leaflet; (B) Apical four-chamber view, showing multiple echogenic structures attached to the posterior mitral valve leaflet; (C) Apical two-chamber view with colour flow Doppler showing mitral regurgitation.

**Figure 2:**
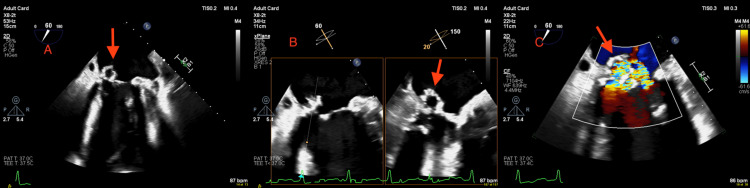
Transesophageal echocardiogram (A) Mid-oesophageal two-chamber view showing formation of PMVL aneurysm; (B) X plane assessment of PMVL showing aneurysm formation; (C) Colour Doppler assessment of MV showing MR due to perforation of mitral valve aneurysm. PMVL: posterior mitral valve leaflet; MR: mitral regurgitation; MV: mitral valve

A transthoracic echocardiogram (TTE) was performed in the context of fluid overload, raised brain natriuretic peptide (BNP) >35,000, and a history of previous positive blood cultures (*S. Aureus*). It showed multiple echogenic structures attached to the posterior mitral valve leaflet, raising suspicion of vegetations. Mild to moderate mitral regurgitation (MR) was seen along with a calcified posterior mitral valve leaflet (PMVL), P2 scallop aneurysm, and suspected perforation (Figure 1). The left ventricular ejection fraction was 40-45%. Intravenous flucloxacillin was commenced for suspected *S. aureus* IE. A TEE was performed, which confirmed the presence of a calcified PMVL with an aneurysm along with perforation, resulting in severe MR, likely due to IE (Figure 2).

Diagnosis of IE due to *S. aureus* with severe MR secondary to a perforated mitral valve aneurysm was confirmed as per the major Duke Criteria of Definite Infective Endocarditis [6,7]. Assessment by the cardiothoracic team suggested medical treatment for IE with a course of antibiotics for six to eight weeks, with future consideration of surgery if functional status improves. Due to multiple comorbidities and an unfavourable risk-benefit profile, surgical intervention was deemed unfavourable. 

The patient was discharged with a plan to complete a course of antibiotics with the Outpatient Parental Antimicrobial Therapy (OPAT) team and regular review by district nurses and IE MDT follow-up. BNP gradually improved with diuresis. Serum creatinine fluctuated within dialysis-adjusted ranges during hospitalization. The patient completed the course with regular review by district nurses and the IE multidisciplinary team (MDT), and remained clinically stable with no recurrence of bacteremia.

## Discussion

MVA is a saccular outpouching of the mitral leaflet, with the anterior leaflet being more commonly involved than the posterior leaflet and reported incidence of 0.2-0.29% [8,9]. The exact underlying mechanism is not known. Various cardiac conditions and non-cardiac causes have been associated with the formation of aneurysms. However, degenerative changes with aging may render weakening of the mitral valve, bringing about aneurysm formation and its perforation [10]. Although MVA may occur in congenital or connective tissue disorders, IE remains the predominant etiology in the current literature, especially with *S. aureus* [11]. Large aneurysms are more likely to rupture than small ones; whereas, size has no association with the incidence of perforation [12].

IE is a fatal disease with a mortality rate of 20-25% and an annual incidence of 1.5/100000-15/100000 [13]. It is an infection of the endocardium most commonly affecting the aortic and mitral valves, whereas involvement of the tricuspid and pulmonary valves is seen in less than 10% of cases [14]. Subacute endocarditis is characterised by a gradual onset and a lengthy course lasting between three weeks and six months. Due to the non-specific clinical manifestation and unexpected course, delayed diagnosis may have an adverse outcome [15]. Therefore, prompt diagnosis and timely management are the mainstay in the treatment of these patients.

*S. aureus* is the leading cause of blood-borne infections like sepsis and endocarditis worldwide [16]. Local skin infections may serve as an entry point for pathogens into the bloodstream. In our patient, the infection was probably caused by gangrene, and the underlying previous history of diabetes and renal failure made the immune system susceptible to infections. The virulence of *S. aureus* is attributed to its capsule, cell wall composition, and formation of extracellular invasive enzymes and toxins, making tissue invasion feasible, facilitating survival within phagocytes, unique host-pathogen interactions, and the capacity to develop resistance to antimicrobials [17,18]. Due to its high virulence, *S. aureus* is characterised by rapid progression and destructive cardiac lesions, such as perforation in our case.

Echocardiography is the primary investigation for establishing the diagnosis of IE. It is used to assess disease severity, cardiac function, and cardiac complications. Recent advancements in ultrasound technology allow segmental MV analysis of three-dimensional (3D) echocardiographic data sets, facilitating accurate diagnosis [19].

Real-time 3D TEE has been shown to provide spatial configuration of cardiac structures and their anomalies and is considered superior to TTE in diagnosing complex cardiac lesions [20]. In our case, TTE identified suspicious vegetations and MR, but TEE, particularly 3D TEE, provided definitive characterization of the aneurysm, perforation, and P2 scallop involvement, thereby guiding the MDT’s management decision.

Patients diagnosed with IE are treated with empirical therapy followed by culture-sensitive antibiotics. Most cases are treated with beta-lactam agents, and cefazolin or vancomycin is used in those who are hypersensitive to penicillin. Blood cultures must be drawn every 24-48 hours after the initiation of antibiotics and continued until they are negative [21]. Conservative management in the presence of complications is reserved for those cases where surgery is very high risk, as mentioned in the current case.

## Conclusions

MVA is an uncommon condition, resembling mitral valve prolapse or regurgitation clinically, and may occur as an isolated pathology. IE caused by S. aureus is highly virulent, frequently resulting in complex, destructive tissue lesions, such as the perforation observed in this case. TEE is an advanced imaging modality that facilitates the diagnosis of complex cardiac lesions. Our study highlights the diagnostic capability of 3D TEE, which enables accurate characterisation of the shape and location of valvular perforation. Management of IE must be individualised, and the optimal outcome depends on prompt diagnosis, multidisciplinary decision making, and early treatment, including timely intervention.
